# Modeling climate change adaptation for sustainable coastal zones using GIS and AHP

**DOI:** 10.1007/s10661-023-12287-2

**Published:** 2024-01-15

**Authors:** Mohamed Marzouk, Shimaa Azab

**Affiliations:** 1 Structural Engineering Department, Faculty of Engineering, Cairo University, Giza, Egypt; 2 Environmental Planning and Development Center, Institute of National Planning, (INP), Cairo, Egypt

**Keywords:** Climate change, Coastal vulnerability, Adaptation systems, Geographic information system, Decision support systems

## Abstract

The world is currently confronting one of its biggest environmental challenges: combating climate change. Coastal zones are one of the areas thought to be most sensitive to current and future climate change threats. The paper integrates Remote Sensing (RS), Geographic Information System (GIS) techniques, and Multi-Criteria Decision Analysis (MCDA) to detect vulnerable areas from climate change impacts in coastal zones in order to recommend adaptation systems in new coastal zones that can withstand various climatic changes. The proposed decision-making framework was developed in three phases: 1) climate data collection and processing; 2) Coastal Climate Impact Assessment (CCIA) model development; and 3) implementation and adaptation system selection. The climate data collection and processing phase involves determining the most significant climate change parameters and their indicators that affect coastal zone stability, extracting climatic data indicators from different climate database sources, and prioritizing the selected indicators. The indicators’ weights were estimated using the Analytical Hierarchy Process (AHP) through a questionnaire survey shared with experts in climate change impacts. A CCIA model development phase involves the formulation of the proposed model using GIS technique to discover the vulnerable areas according to the most dominant impact. The implementation and adaptation system selection phase involves the application of the framework to Al-Alamein New City in Egypt. A sensitivity analysis was conducted to measure the behavior of several climate change parameters to identify the most critical parameter for climate change in Al-Alamein New City. The results showed that the geology of the region is the most crucial component influenced by climate change. It is capable of producing a very sensitive area in the coastal zone while also taking other factors into account. When creating new urban neighborhoods, the erosion of the shoreline is the least important factor to consider. This is because coastal deterioration is caused by both the influence of metrological data on the region and the impact of human activity. Shoreline deterioration will be reduced if climate conditions are maintained while limiting the impact of human activities. To adapt to the long-term effects of climate change on coastal zones, a combination of soft and hard protection systems should be considered.

## Introduction

Climatic change is a continuous process that has happened since the first stable global climate system was formed and the first living place on Earth was developed (IPCC, 2013). Governments have agreed to move towards better sustainability through the Paris climate change agreement and the 2030 Agenda for Sustainable Development. Most recently, numerous nations made commitments to achieve net-zero greenhouse gas (GHG) emissions by the middle of the century. To achieve these commitments, long-term goals must be defined in order to balance present needs with those of the future. These goals serve as a solid foundation for tracking the progress made by each government. With increasing poverty and a lack of economic resources in developing countries compared to developed countries, developing countries are most vulnerable to climate change. Most developing countries suffer from immense challenges due to climate extremes such as heat waves, torrential rain, freezing temperatures, tropical cyclones, and floods. This is especially true in countries that have a high population density, limited resources, a reliance on non-renewable energy sources, and little ability to respond to these environmental threats (Rahman, 2018).

Coastal zones are considered one of the areas with the densest populations and essential infrastructure due to their complex socio-economic systems. The majority of the world's population lives in these zones (Nelson, 2018). They occupy 20% of the land area and house 40% of the world's population (Rangel-Buitrago et al., 2020). Despite this significance, these zones are particularly vulnerable to global environmental changes, such as climate change. Climate change will pose threats to different components of coastal zones. One of the most serious threats posed by climate change in these zones is sea-level rise. Where about 7% of the world's population lives in flood-prone areas (Li et al., 2009). Increasing sea levels will lead to saltwater intrusion on land, coastal erosion, and exacerbated the effect of coastal storms (Woodruff et al., 2018). This leads to significant socio-economic effects such as population displacement, loss of properties, recession of economic and industrial activities, loss of coastal habitats, and loss of tourism, the recreation, and transportation functions (Torresan et al., 2008). In addition, increased temperatures will result in more heat waves, which will have an impact on human and ecosystem health, putting humans, animals, and plants in danger of illness and even death (CoastAdapt, 2017).

Preserving coastal zones from the effects of climate change is a major challenge due to changing coastal conditions. In the past, when it came to development-related consequences on the coast, planners and managers would often create concrete barriers and shields to safeguard human settlements (French, 2004). However, due to variances in local geomorphic properties and changes in such qualities over time, these adaptation systems have often proven ineffective in the long run. The notion of 'integrated management' was born out of the necessity for long-term solutions to coastal management difficulties as well as a better understanding of dynamic coastal processes (McFadden, 2007). Measuring coastal vulnerability is essential in planning and managing coastal zones (Baučić et al., 2019). The vulnerability was described as “a nation's ability to cope with the repercussions of accelerated sea-level rise and other coastal effects of global climate change” (IPCC, 1992). Vulnerability assessment attempts to categorize coastlines into units with comparable features, establish the form of vulnerability (e.g., erosion vs. inundation), and offer a rating of probable coastline alterations. These assessments are often stated quantitatively, which frequently takes into account five categories (very low, low, medium, high, and very high) to simplify complicated and interacting factors that are utilized to influence coastal management. Coastal vulnerability to Sea Level Rise (SLR), wave erosion, and human effects has been the focus of several assessments (VanZomeren & Aeevedo-Mackey, 2019). Therefore, vulnerability analysis is a reasonable approach for assessing such crises because the information provided may be useful for policymakers to aid in the adaptation processes.

Coastal zones can be protected from climate change impacts in two ways: mitigation and adaptation. Mitigation is an intervention to reduce GHG emissions sources or improve GHG sinks (IPCC, 2001). In comparison, adaptation is the adjustment process to the actual or expected climate and its effects to moderate harm or take advantage of beneficial opportunities (IPCC, 2014). It is determined according to the country’s economic position as well as the results of a vulnerability assessment based on the geographical location, the types of climate change impacts, and the intensity of negative environmental consequences.

Hard engineering protection and soft engineering protection are the two forms of coastal adaptation management. Hard engineering protection is man-made structures that protect the shoreline from extreme and destructive natural processes. These structures help safeguard coasts by absorbing wave energy and reducing erosion and floods. These structures are costly, short-term solutions that frequently have a harmful influence on the environment, might have negative consequences farther down the coast, and diminish the ability of the coastline to respond naturally to changing conditions (Luo et al., 2015). Appendix 1 illustrates the hard adaptation structure used in coastal zones. On the other hand, soft engineering protection works with nature rather than against it to safeguard the shoreline. It employs ecological principles and practices, resulting in a lower negative influence on the natural environment. It is less expensive to develop and maintain than hard engineering projects and offers more long-term, sustainable solutions (Bongarts Lebbe et al., 2021). Appendix 2 presents the soft adaptation structure used in coastal zones. Therefore, this paper presents the development of a decision-making framework for assessing climate change impacts on coastal vulnerability and adopting a climate change adaptation protection system to coastal zones, overcoming existing limitations of currently utilized protections such as downdraft erosion and disrupting natural processes.

Remote sensing (RS) time series are the main part of the tracking system for climate variability and changes (Allard, 2017). RS and Geographic Information System (GIS) might be combined to accomplish these goals. GIS provides a platform for organizing thematic maps in several forms (e.g., raster or vector data) and is used to execute logical and mathematical calculations during vulnerability assessments. GIS is recognized as a decision-support system that integrates geographically referenced data in a problem-solving context (Cowen, 1990). It combines many types of data into accessible formats, evaluates and analyzes data, and provides descriptive and predictive modeling of various scenarios. Mitigating the consequences of disasters such as climate change necessitates real-time access to disaster-related information. The susceptibility of coastal zones may be recognized using environmental satellites by monitoring the present situation—before, during, and after a catastrophe. GIS methods provide an appropriate framework for integrating and evaluating the various data sources needed for climate change monitoring. A decision support system (DSS) is used to aid in decision-making, judgment, and action. A DSS, besides GIS and RS, is responsible for filtering, and analyzing massive amounts of data, generating precise information that may be used to solve problems and make choices. It is critical to describe the temporal and spatial evolution of climate change vulnerability and its driving factors in order to cope with it (Jiang et al., 2021).

The Analytic Hierarchy Process (AHP) is one of the MCDA techniques to detect the importance and relative weight. Several MCDA approaches have been used to assess coastal vulnerability due to environmental and human hazards. It is a decision-making tool that is used to provide a reasonable framework for making an informed decision, considering various indicators or criteria while prioritizing and selecting (quantifying) choices (Saaty, 1977). Bagheri et al. (2021) used AHP for coastal city hazard management, and Maanan et al. (2018) assigned AHP and principal component analysis (PCA) to help them assess coastal vulnerability to environmental hazards. While Boulomytis et al. (2015) utilized Compromise Programming (CP) to evaluate the watershed’s vulnerability to floods in a future scenario, the AHP is the most commonly used and broadest process among the MCDA techniques (Bagheri, et al., 2013; Yannis et al., 2020).

## Literature review

Several research efforts have been devoted to assessing coastal vulnerability to climate change impacts using different methodologies. Satta (2014) established an Index-based method for the integrated analysis of coastal risk to multiple hazards that considers the impacts of SLR, storm variability, and human-induced forcing. The author identified population increase and tourism development as the most significant human-induced causes, with coastal erosion, coastal flooding, and seawater intrusion as the most significant natural hazards in Mediterranean coastal zones. The study produced vulnerability and risk maps for every single hazard and multiple hazards .Sanuy et al. (2018) applied the Bayesian network-based approach to evaluate coastal risk by predicting risks at the receptor scale, which were converted into impacts through vulnerability relations. The proposed framework has helped analyze storm-induced risks and strategies significantly. Haugen et al. (2018) proposed a generic framework to monitor climate change impacts on historic buildings and their interiors to build a data-driven decision-making process. The study asserted that effective monitoring could be achieved by systematically using more detailed images.

Sahoo and Bhaskaran (2018) developed a study to investigate the physical, environmental, social, and economic impacts on coastal vulnerability associated with tropical cyclones. Weather parameters, along with storm surge height and onshore inundation, were used to estimate the Physical Vulnerability Index (PVI) using the GIS-based approach. El-Shahat et al. (2021) evaluated the vulnerability of African coasts to SLR. The authors applied the Coastal Vulnerability Index (CVI) method using GIS and the RS approach. The study has customized CVI to include seventeen parameters that represent the physical, social, and economic features of coastal zones. Vieira et al. (2021) used GIS and RS to assess coastal erosion from climate change, taking into consideration seven factors: elevation, geomorphology, geology, land cover, anthropogenic activities, distance to the shoreline, and maximum tidal range. The authors considered the CVI method for vulnerability to erosion evaluation. Rahmawan et al. (2022) examined six parameters (geomorphology, elevation and slope, land use, rigid structure, and coastline changes) to evaluate coastal vulnerability using GIS-based and scoring assessments. The coastline change is quantified based on the Modified Normalized Difference Water Index (MNDWI). The scored parameters are then analyzed using CVI. Palacios-Abrantes et al. (2022) developed the Nature Futures Framework (NFF) by the Task Force on Scenarios and Models of the Intergovernmental Science-Policy Platform on Biodiversity and Ecosystem Services as a heuristic method. The authors concluded that a variety of different viewpoints on people's attitudes toward the environment might lead to decision-making that is adaptable and resilient in dealing with the effects of climate change on social-ecological systems.

El-Masry et al. (2022) investigated the potential effects of climate change and sea-level rise on the vulnerability of the Mediterranean coastal of the El Hammam—El Alamein. The assessment of the area's and coastal tourism's vulnerability to climate change and SLR through the development of a digital elevation model (DEM) and inundation models, as well as the assessment of temperature change using the tourist climate index. According to the study, most seaside resorts will be submerged. Roy et al. (2023) investigated the vulnerability of the potential impact of climate change on SLR and coastal habitats based on the CVI method. Their study characterizes the physical setting, including geomorphology (G), sea level change (SLC), coastal slope (CS), relative sea-level change (RSLC), mean wave height (MWH), mean tide range (MTR), shoreline change rate (SCR), land use and human activities (LU), and population (P). The study concluded that vulnerability assessment can help the decision-maker for consider the most appropriate development strategies to maintain the sustainable development of coastal ecology.

Prior literature efforts analyzed coastal zone vulnerability to climate change. However, such efforts did not consider the results of hard protection systems on the dynamic relationship between climate change indicators in their natural state (Cabana et al., 2023; Gargiulo et al., 2020; Wei et al., 2021). Also, previous research studies lack the representation of weights using MCDA approaches such as the AHP for climate change indicators to evaluate, rank, and define the relative weights and relevance of each climatic criterion (Allipour Birgani et al., 2022; Thirumurthy et al., 2022). Most aggregated climate-change vulnerability scores using the classic MCDM approach such as a weighted sum method (WSM) for key indicators (Kim & Chung, 2013). The spatial influence of all indicators affecting the shoreline, local weather, topographical structure representing the earth's form, and zone geology in defining the vulnerable areas due to climatic changes was not considered. The vulnerability of coastal zones to be suitable for the construction of sustainable new communities was not studied as a result of the effect of each parameter separately, and the comparison between these results and alternative scenarios for other parameters was not taken into consideration. Previous research studies did not investigate the sensitivity of relative weights and degrees of importance of variables in transforming the form, area, and locations of coastal zone vulnerability (Azab, 2022).

The hypothesis of this paper is that climate change impacts have negative effects on any coastal zones, and these effects are represented in threatened areas with different degrees of vulnerability. To achieve the desired goal and verify the paper hypothesis, this research proposes a decision-making framework capable of evaluating the impact of climatic change on the vulnerability of coastal zones. This aids in adopting a protection system for climate change in coastal communities. The proposed framework contributes to resolving the issue of recognizing the harmful impacts of climate change on coastal zones and maintaining their long-term survival. Decision-makers will benefit from this framework and its findings as they choose the most suitable solutions to climate change adaptation. The framework combines RS, GIS, and MCDA analysis to plan new sustainable coastal communities that can resist various climatic changes. Considering the above clarification of climate change problems in coastal zones, systems to address them, and the gaps in previous studies, this paper attempt, through the proposed framework, to answer the question, do the climate change indicators have the same effect in the formation of vulnerability areas of their different degrees?

## Proposed decision-making framework

**Fig. 1:**
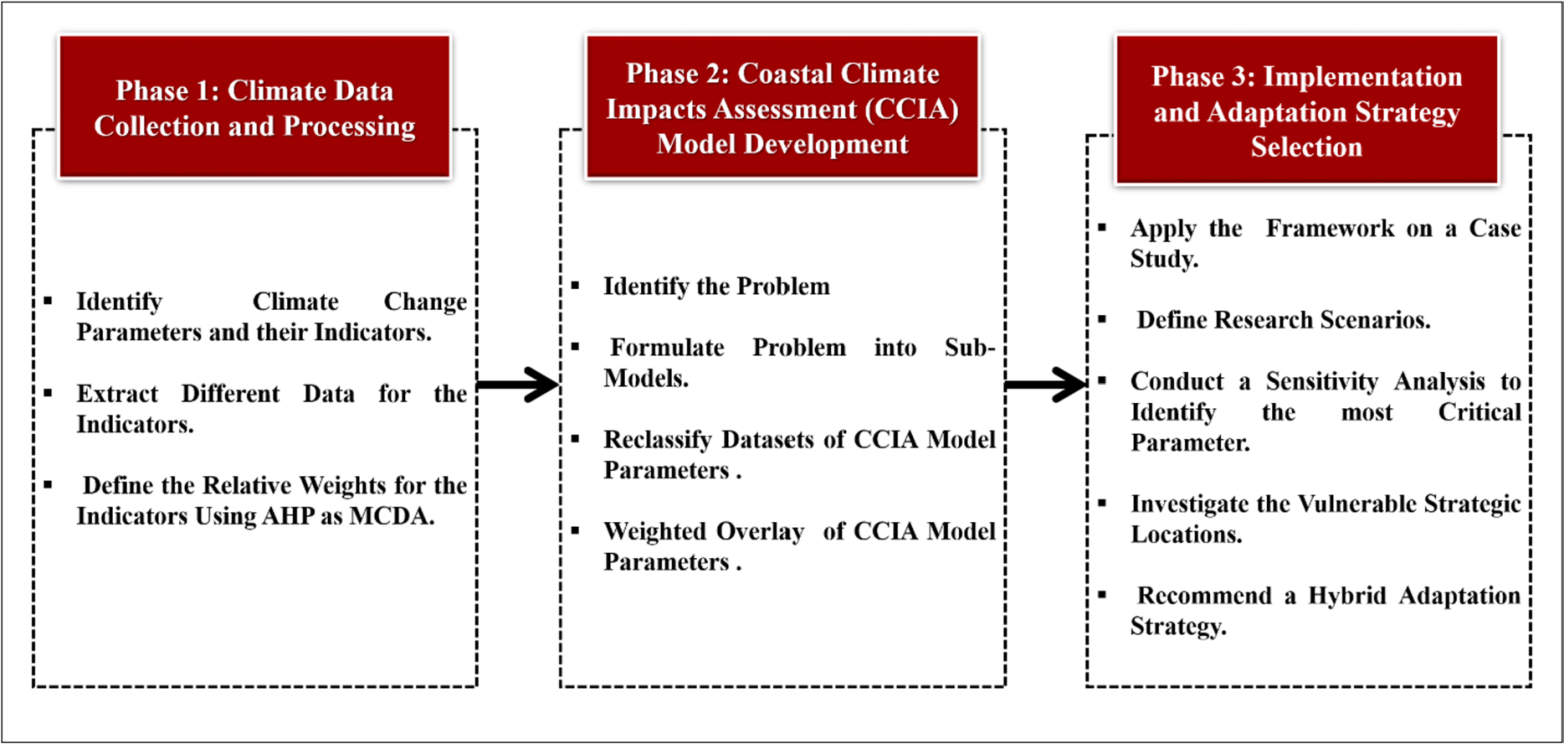
Proposed coastal adaptation framework

The developed framework helps government agencies and/or contractors integrate RS, GIS, and MCDA. It has great potential for coastal vulnerability assessment to provide an accurate indication of climate change's impact on the dynamic environmental systems of coastal zones. This leads to choosing the most suitable coastal adaptation system to resist climate change impacts and reduce coastal vulnerability to current and projected climate changes. RS and GIS are important for generating baseline data on land cover shifts in coastal zones. They are used to capture, explore, manage, and model all data forms that are geographically referenced. The integration of RS and GIS techniques helps to generate a descriptive and predictive Coastal Climate Impact Assessment (CCIA) model for more accurate monitoring and decision-making. The proposed framework’s application follows a procedure consisting of three phases: climate data collection and processing, CCIA model development, and implementation and adaptation system selection (see Fig. 1).

### Climate data collection and processing

Identification of the most significant climate change indicators that impact the stability of coastal zones is performed in three steps. The first step concerns identifying the main indicators of climate change according to the literature. To evaluate the effect of SLR in coastal zones, the sea level pressure, the atmospheric pressure at sea level, is used to indicate the sea level. The behavior of sea level changes by changing the value of sea level pressure as the inverse barometer effect, as the higher the pressure, the lower the sea level, and vice versa. (Wunsch & Stammer, 1997). Marzouk et al. (2021) identified the most affected climate change indicators on the suitability of coastal zones, which are divided into four main effective groups: meteorological, topographical structures (Earth Shape), engineering geology, and shoreline parameters (see Table 1).

**Table 1:** Considered climate change indicators

Parameters	Indicators	Source of Data	Unit
Meteorological Data	Surface Temperature	NOAA (2019)	Celsius
	Precipitation		mm
	Sea Level Pressure		mm of mercury (mmHg)
	Dew Point		Celsius
	Wind Speed	NASA (2019)	m/sec
	Wind Direction		from 0° to 360°
Topographical Structure	Slope	Digital Elevation Model (DEM)	Degrees for the inclination of a slope
	Coastal Regional Elevation		m
Engineering Geology	Composition of the Earth	Geologic Map (NARSS, 2007)	Type
Shoreline	Erosion	Land Use Classification (Satellite Imagery)	km^2^
	Accretion		km^2^

The second step is extracting data for each identified indicator based on the studied area of the research from various climate database sources, such as RS sensors and climate stations. RS is able to sense, map, and monitor changes in the coastline because they are recognized as main sources of trustworthy and durable data for all circumstances needing spatial data at a variety of temporal and geographical scales. The Advanced Spaceborne Thermal Emission and Reflection Radiometer (ASTER) data of Global Digital Elevation Model (GDEM) at a horizontal spatial resolution of 30 m is used to create detailed maps of topographical characteristics such as coastal slope and coastal regional elevation. A geologic map is used to study engineering geology, or the composition of the earth’s components. Finally, climatic data are gathered from two climate databases based on daily observations.

The final step involves developing a scaling questionnaire and decision-making model from climate change indicators to evaluate, rank, and define each indicator’s relative weights and importance for performing a realistic representation of weights. The AHP model has been developed as an MCDA technique for pairwise comparison to determine priorities and relative weights of indicators.

### Analytic Hierarchy Process (AHP) model

The relative importance of the indicators and their weights must be determined to integrate all of the parameters and their indicators into a single spatial model. This is deemed important to determine the vulnerability areas due to climate change in coastal zones to promote a suitable development for new communities and determine the most influencing parameter. This paper uses the AHP as one of the MCDA techniques for prioritizing the various indictor and ranking them as per their weights. This is according to a questionnaire survey focusing on expert knowledge about the impacts of climate change and weather conditions to obtain a preference degree for each impact of climate change according to the following procedure:

### Data collection using questionnaire experts survey

**Fig. 2:**
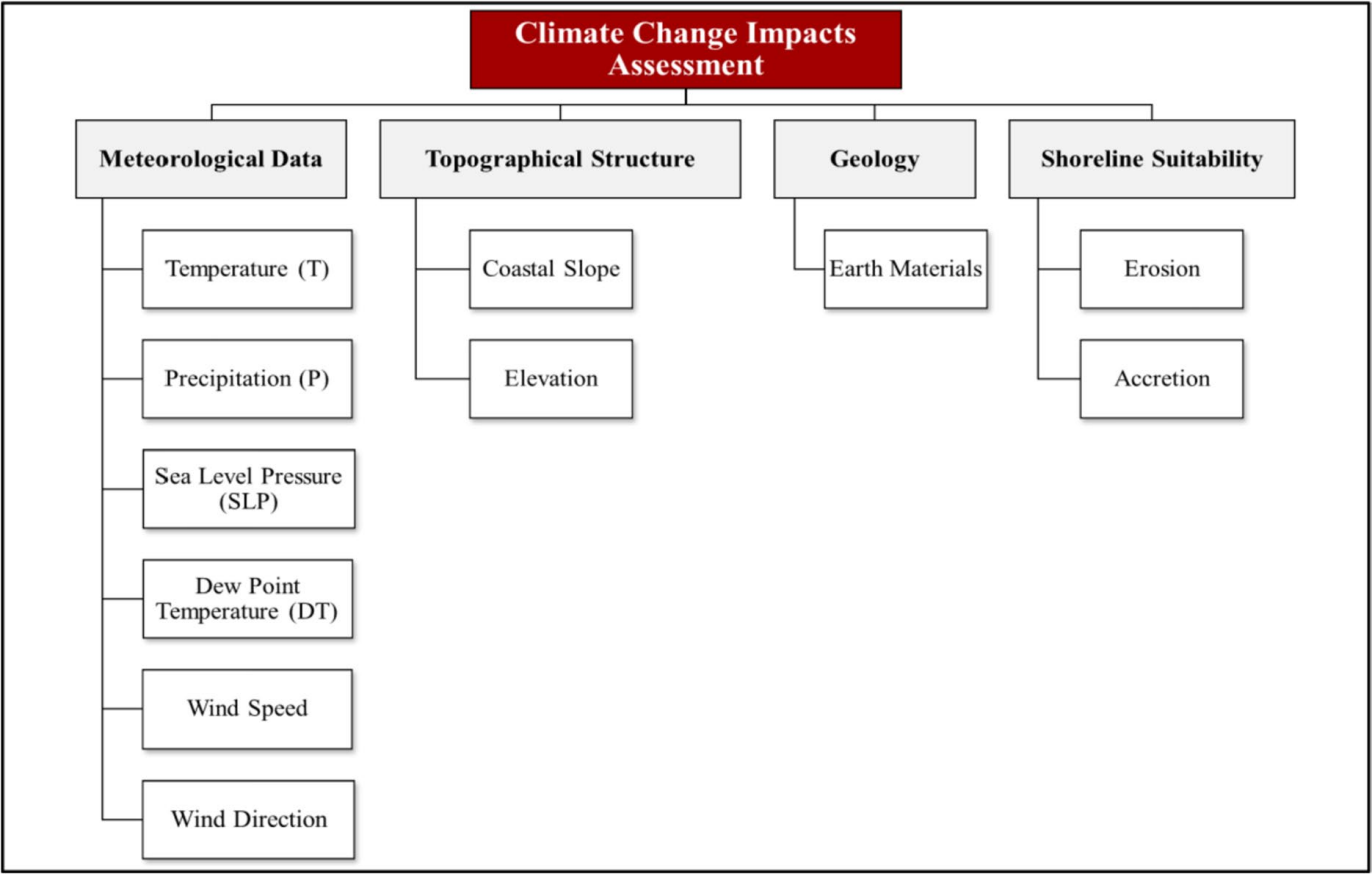
Designated AHP for climate change impacts

The AHP technique represents a problem and then develops priority scales for gauging qualitative performance using numeric scale calibration (Saaty, 1977). The first stage in the AHP approach is to break down the problem into smaller components, starting with the goal, which is the climate change impacts assessment, which is decomposed into research parameters, and finally the indicators. The four main parameters of this paper with 11 indicators are meteorological data; topographical structure (earth shape); engineering geology; and shoreline suitability (see Fig. 2).

The questionnaire was distributed and collected within a two-week timeframe. The questionnaire is divided into four sections: basic information, introduction, informed consent form, and questions about the level of importance of various factors (i.e., the importance degree of Factor 1 (F1) when compared to Factor 2 (F2) according to A Saaty’s scale) (Saaty, 1985). The scale is used to form pairwise comparison matrices at all levels. For example, the pairwise comparison matrix in the first level includes meteorological data, topographical structure (earth shape), engineering geology, and shoreline suitability. The expert choice software estimates the weights of the parameters’ indicators. The questionnaires were administered online and face-to-face to a diverse group of climate specialists. Fifteen Egyptian experts and specialists have more than 15 years of experience, according to their responses from various governmental agencies and authorities, such as the National Authority for Remote Sensing and Space Sciences (NARSS), Ministry of Environment, Egyptian Environmental Affairs Agency (EEAA), National Research Center, Faculty of Urban and Regional Planning, Egyptian Meteorological Authority (EMA), National Institute of Oceanography and Fisheries, Ministry of Water Resources and Irrigation, and Institute of National Planning (INP).

### Coastal climate impact assessment model development

**Fig. 3:**
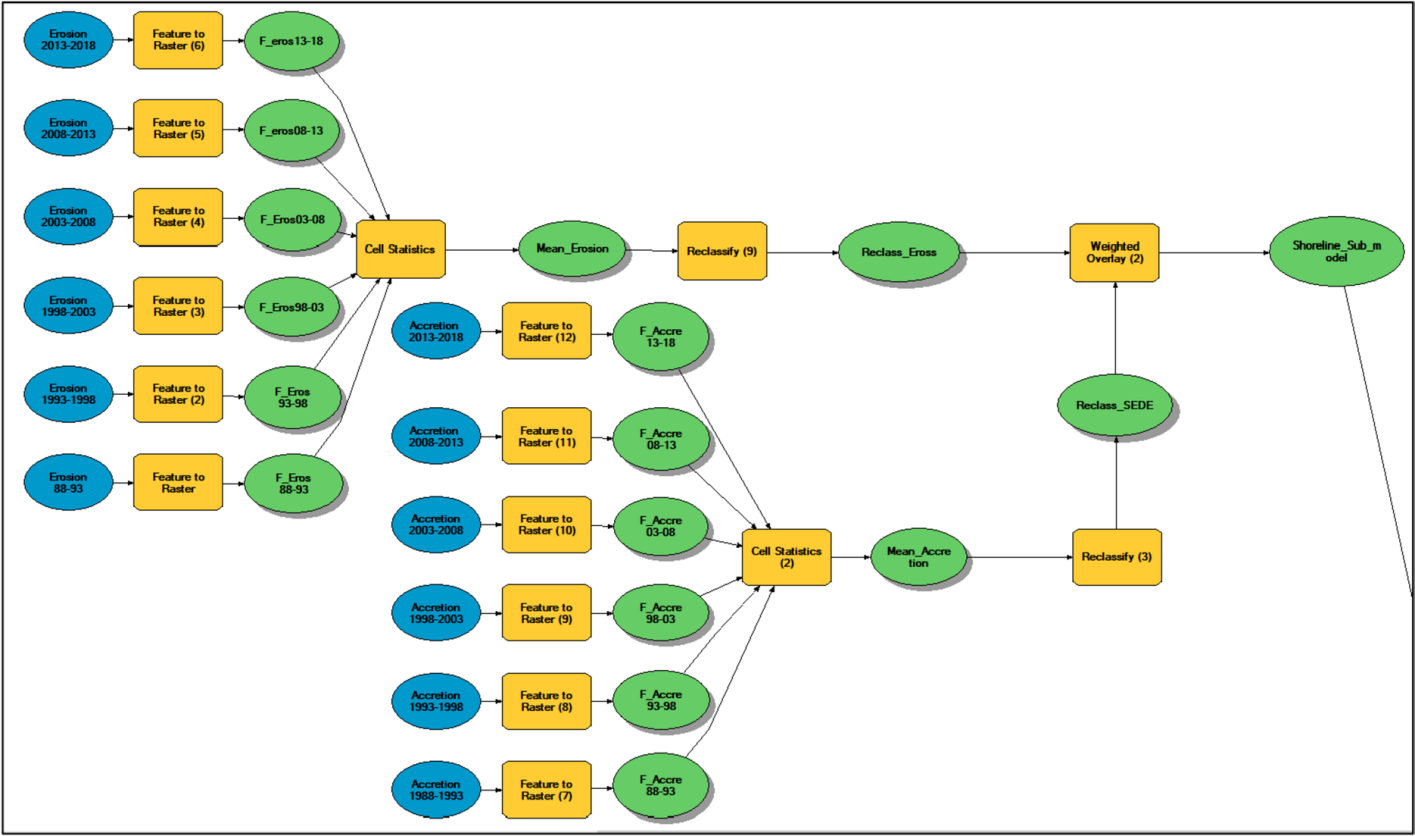
Shoreline sub-model from CCIA model

**Fig. 4:**
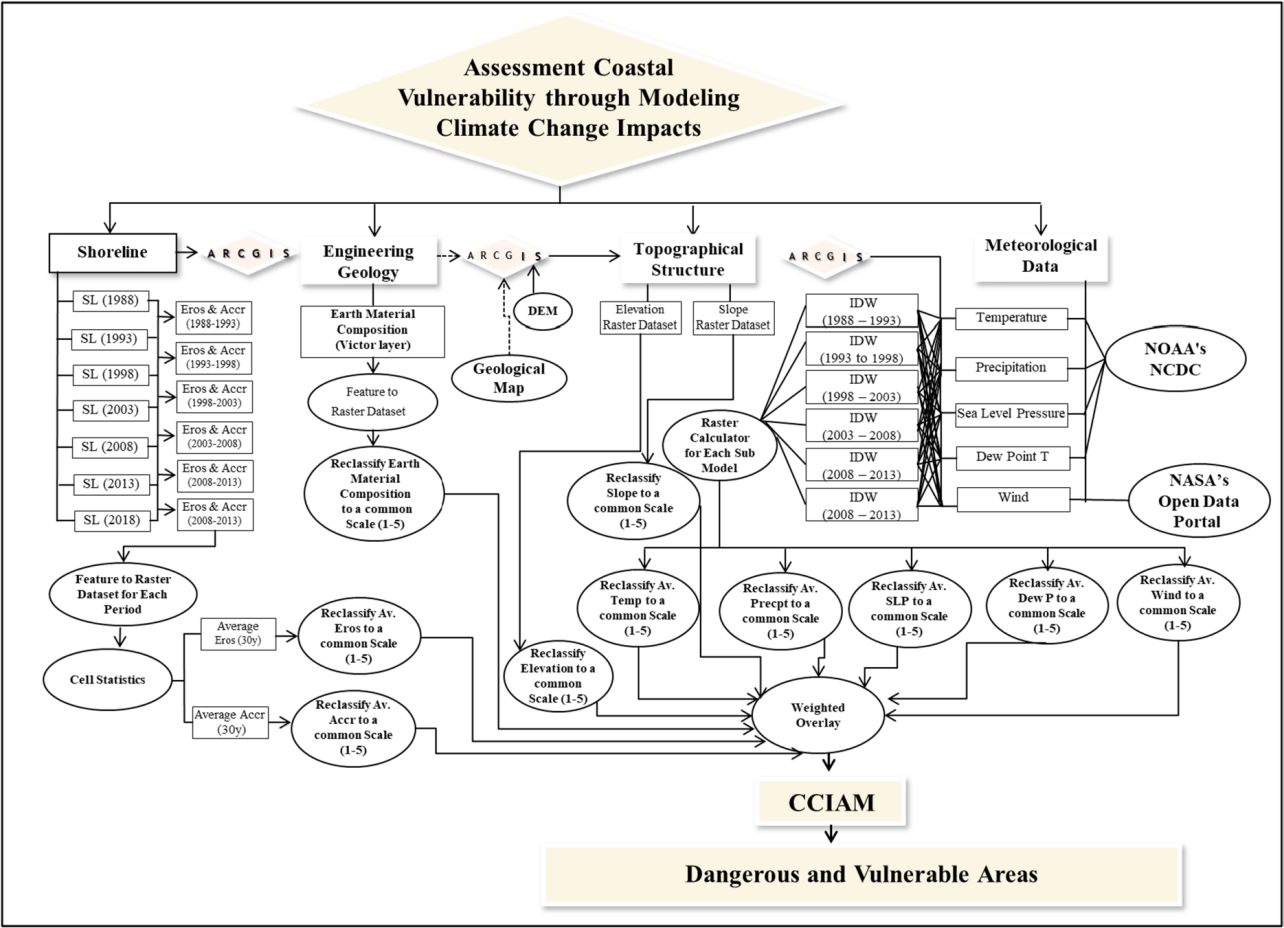
The procedure of formulating and analyzing the proposed CCIA model

A CCIA model is developed using RS and GIS techniques. A more in-depth explanation of the CCIA model may be found somewhere (Azab, 2022; Marzouk et al., 2021). The CCIA model has been developed according to four steps:

- Step 1: Modeling the problem
  This step is very important in modeling the climate indicators data, which aims to specify all aspects of the problem, such as defining the objectives, time horizon, and decision variables. The study period is taken to be 30 years, according to the World Meteorological Organization (2017). It is divided into equal periods (i.e., 5 years for each period) starting in 1988 and ending in 2018 to monitor and predict changes to the climate.
- Step 2: Formulation problem sub-models
  This step aims to convert the study problem into sub-models and perform the necessary processing of each climate change indicator using a GIS environment. Each sub-model is manipulated in the GIS environment before being integrated into a single model. To run in the model, all sub-models are turned into raster datasets. Each layer or point must be turned into a raster dataset before being integrated into a single model. For example, Fig. 3 depicts the shoreline sub-model from the CCIA model. It includes two sub-models: erosion and accretion. The output is the rate of shoreline change (positive for accretion and negative for erosion from land).
- Step 3: Reclassify datasets
  The third stage is to reclassify the values of each sub-model of a sequence of input vector data to a common scale, assigning maximum numbers to the most harmful characteristics and lower values to the least harmful attributes.
- Step 4: Weighted overlay
  The fourth step is a weighted overlay analysis of all sub-models in the map combination according to the importance degree of each sub-mode. This enables the computation of a multiple-criteria analysis between many imagery rasters and the control of the effect of various indicators in the suitability model. Each sub-model has a proportional impact on the total assessment based on how important it is. All sub-models are equally weighted at 100%. For the importance degree of each sub-model, the relative weights of sub-models obtained from the AHP approach are incorporated into a GIS to generate the vulnerable areas or hotspots most susceptible to climate change. Also, this paper uses equal weights for all sub-models as a base scenario to examine each parameter’s effects that constitute the vulnerable areas. The procedure for formulating and analyzing the proposed CCIA model is depicted in Fig. 4.

### Implementation and adaptation system selection

This phase is concerned with controlling the consequences of climate change and adapting the ecological system in response to real or anticipated climatic stimuli and their effects or implications. Validation of the developed model’s performance is necessary to build confidence in the proposed CCIA model. In this phase, a real-life case study is worked out to demonstrate the use of the proposed CCIA model.

A sensitivity analysis is conducted to choose the most critical parameters affected by climate change that have the potential to form the most vulnerable areas. It provides an MCDA model for describing the uncertainty linked to the effects of climate change indicators. Sensitive Analysis is a quantitative model to assist decision-makers in their decision-making processes. An appropriate adaptation system should be chosen to develop new sustainable communities in coastal zones. The challenge of coastal adaptation to climate change necessitates establishing a primary goal that can influence decision-making. The basic goal of the challenge is to define the most critical parameter influenced by climate change, which has the potential to weaken coastal communities if not taken into account.

The investigation of the affected degree of climate change parameters to detect the riskiest areas in the coastal zone is achieved through testing the sensitivity of critical parameters. It is conducted by considering different weights for the parameters and their indicators, forming different scenarios. These scenarios are simulated using the CCIA model. Six scenarios are devised to examine the behavior of each indicator and parameter in the final output. Comparative analysis is performed using a GIS environment to explore the vulnerable strategic locations in the study area, which match the weak spots inferred from the case study. After conducting these tests, decision-makers can decide on the best scenario for the riskiest areas. Finally, it suggests an adaptation system for the study area towards climate change.

## CCIA model implementation

**Fig. 5:**
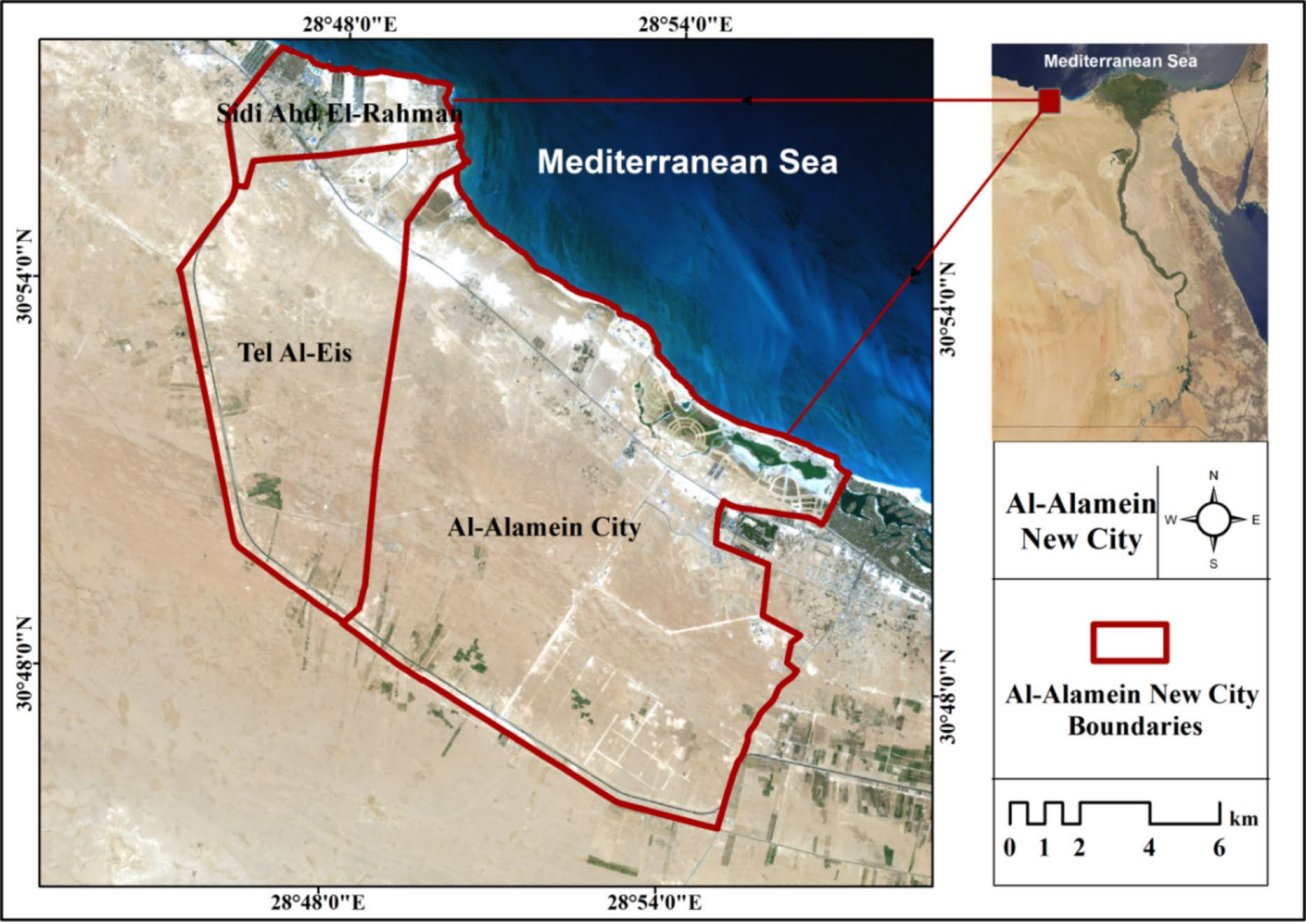
Study area map of Al Alamein New City in Egypt

The proposed CCIA model is conducted in Al Alamein New City in northern Egypt. Al Alamein New City is located on the North Coast between latitudes of 30° 49′ 48.25" N and longitudes of 28° 57′ 18.07" E. It is located within the geographical limits of the governorate of Marsa Matrouh. It covers an area of approximately 227.65 km^2^. It is being developed to be a residential and tourist city. In addition, it contributes to reducing the problem of unemployment by providing 1.5 million job opportunities (Associated Consultants, 2015). It is divided into three divisions based on the administrative borders established by the Central Agency for Public Mobilization and Statistics (CAPMAS), as seen in Fig. 5. The first sector is Sidi Abd El-Rahman, which is located in the city's west and accounts for around 6.5% of the overall area. The second sector is Al Alamein City, which is located in the city’s east and accounts for approximately 65.2% of the overall area. The last component is Tel Al-Eis, which is positioned between the two preceding sections and accounts for around 28.3% of the city’s total area. The strategic objectives for developing this new city are as follows: 1) increasing national and regional income; 2) reducing population density in existing cities; 3) offering diverse job opportunities; and 4) creating a livable city where the residents will have an adequate standard of living (e.g., research, logistics, etc.).

As for the implementation of the CCIA model, all indicator datasets have been created and included in the developed CCIA model as a raster layer for each indicator. For shoreline spatial data, seven multispectral Landsat images were downloaded. Pre-processing and processing have been conducted on the extracted images using ENVI 5.1 software. The mosaicking of images was not necessary since the entire study area fell within a single scene. After classification, the shoreline for each image has been extracted, and erosion and accretion have been calculated using ESRI ArcGIS 10.4.1 software. For topographical structure, slope and elevation have been estimated and mapped from ASTER based DEM of Al Alamein New City with 30 m spatial resolution. The data on meteorological indicators: surface air temperature, precipitation, sea level pressure, and dew point temperature, have been extracted from the databases of the Marsa Matrouh climate station from January 1988 to December 2018. The data extracted from climate stations is displayed in the ArcGIS environment in the form of a point for each year, with its value and coordinates. Because climate change is monitored every 5 years in this study, each period for each indicator is represented by only five points.

Different methods can be used for the interpolation values between the available points, whether deterministic or stochastic methods, such as the Thiessen (Voronoi) polygons, the nearest neighbor, the Kriging, and the spline methods. Choosing one method over the other depends on the distribution of sample points and the parameter being studied. For the Thiessen (Voronoi) polygons, it is considered the simplest method, which uses only one point to interpolate the nearest sample location without considering all other points used to interpolate the unknown point. Also, the nearest neighbor method is utilized to determine the nearest neighbor index, which relies on the average distance from each point to its nearest neighboring point (Sibson, 1981). This method can be used with a small number of input variables, but for a large number of variables, the method struggles to produce the output of the interpolated points (Childs, 2004). Kriging is a geo-statistical interpolation method that uses the spatial continuity of the data, relies on the spatial only without considering the actual values, and does not use any of the point values, owing to interpolated values being lower or higher than actual values (Arun, 2013). The spline method depends on the interpolation process of a two-dimensional minimum curvature spline technique. It is useful for reducing the surface curvature, which results in a smooth surface (Hutchinson, 1988). It cannot be used when the spatial characteristics of sample points are close together and have extraordinary contrasts in their values because this method utilizes slope calculations (change over distance) to figure out the final raster surface (Childs, 2004). While the Inverse Distance Weighted (IDW) interpolation method is an exact method to determine the depth values at un-sampled points by averaging the values of sample data points weighted by an inverse function of the distance from the point of interest to the sampled points (Li & Heap, 2008), It does not need to make any assumptions about the input data. It helps to interpolate dense, evenly spaced cell points, such as flat areas with cliffs. Also, it is considered that if the distance between the points increases, the influence of the cell will decrease on the output value (Childs, 2004; Huang et al., 2011). It is used to interpolate different cell values of many phenomena, such as soil, geology, oceanography, and meteorology (Papari & Petkov, 2009).

Therefore, to predict the different values around these points in this research, IDW interpolation is used. According to the location of sensors yearly, weather points have been signed, and the points between these points for each period have been interpolated using the Inverse Distance Weighted (IDW) method. Using the boundaries of Al Alamein new city, the weather maps have been developed inside the city boundaries only as raster images.

For wind speed and direction, Al Alamein new city boundaries are located between four climate stations, which are Al-Alamin, Marsa Matruh, El-Dabaa, and Siwa (as listed in Table 2). The data from four stations has been extracted every 5 years in net CDF file format and then mapped. The IDW technique was used to interpolate the values of wind speed and direction between the four points each time. The interpolated raster for each layer has been approximated for the values of speed and direction. By choosing to create a fishnet based on the resultant IDW, a grid with regular points has been created, where each point in this grid has a speed value and the location represents its direction. Using the boundaries of Al Alamein new city, the wind maps have been developed inside the city boundaries only as raster images.

**Table 2:** Locations of climate stations closed to Al Alamein New City

Station	Latitude	Longitude
Al-Alamin	30.83	28.955
Marsa Matruh	31.353	27.237
El-Dabaa	31.028	28.445
Siwa	29.203	25.52

For the composition of the earth, the geological map of the city has been extracted from the shapefile of Egypt’s geology. This shapefile is converted into a raster map and inserted into the proposed CCIA model.

## Results

### AHP model results

According to the research parameters, weights were allocated to various indicators based on experts’ assessments. They were computed for each of the three parameters’ indicators. A consistency check is required since weights are only valid if they are acquired from consistent or close consistent matrices (Bagheri et al., 2021). The consistency of the judging matrix is determined by examining the total Consistency Ratio (CR) it is based on the values of the greatest eigenvalue (λmax) and the Consistency Index (CI). The results showed that the CR is less than 0.1, as listed in Table 3. This indicates that the matrixes are reasonably consistent and the errors are acceptable. Decision-makers can use the indicators weights for further calculations and analysis.

**Table 3:** AHP weights for climate change indicators

Parameter	Indicators	Rank	Weight (%)	λmax	CI	CR
Meteorological Data	Surface Air Temperature	1	35	6.589	0.117	0.095
	Precipitation	2	23			
	Sea Level Pressure	3	20			
	Dew Point Temperature	4	9			
	Wind Speed	5	8			
	Wind Direction	6	5			
Topographical Structure	Coastal Slope	1	75	2	0.0	0.0
	Coastal Regional Elevation	2	25			
Shoreline Suitability	Coastal Erosion	1	80	2	0.0	0.0
	Coastal Accretion	2	20			

### CCIA model results

The CCIA model was tested with datasets spanning 30 years. The impacts of the study parameters and each indicator in the four assessment parameters were grouped into five classes, from very low to very high, for areas damaged by climate change. According to the results shown in Table 4, the research hypothesis is valid, as the results of the proposed model showed that there is a variation in the degree of vulnerability in the study area.

**Table 4:**
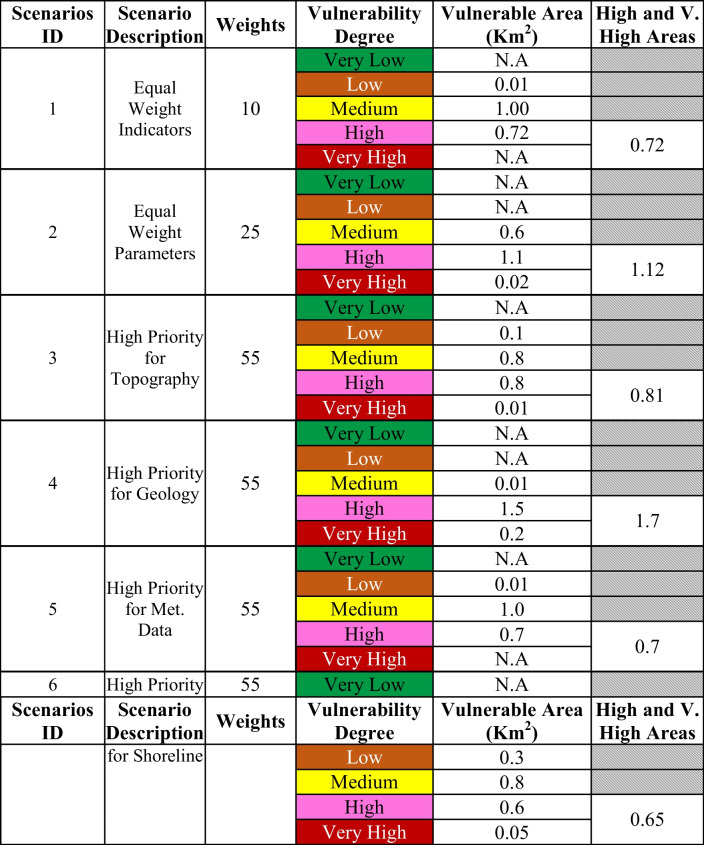
Vulnerability degree of Al Alamein New City scenarios

**Fig. 6:**
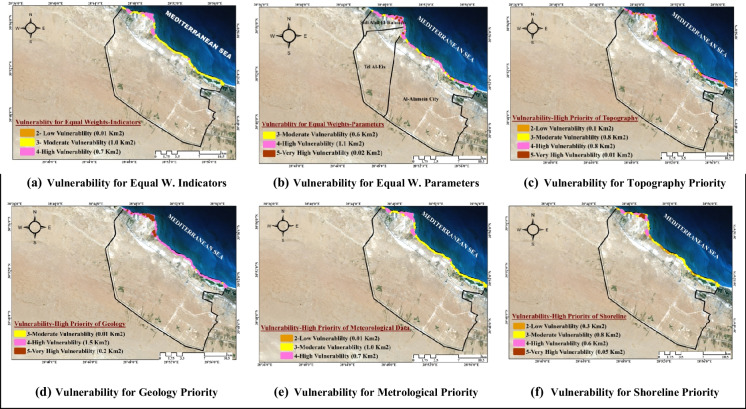
Vulnerable areas maps in Al Alamein New City

Based on the simulated datasets, the results present the sensitivity analysis of climate change impacts on the overall model. Six scenarios are devised to examine the most critical parameters of climate change affecting the study area. These scenarios are as follows: 1) a base-run scenario able to study the model performance under equal weight indicators without dividing them by its parameters, considering equal weight for all; 2) a base-run scenario able to study the model performance under equal weight parameters, considering the relative weights of indicators that were determined using AHP; 3) a topographical structure scenario; 4) a shoreline scenario; 5) a meteorological data scenario; and 6) a geology of a city scenario. Finally, the most critical scenario is compared to the general strategic plan of Al Alamein New City to recommend the critical places that must be adapted to climatic changes. Figure 6 illustrates the output of the overall model according to the six simulated scenarios. The estimated vulnerability degrees of six scenarios are shown in Table 4. The outcomes of six scenarios show that the shoreline is where the consequences of climate change are most noticeable.

In the first scenario, the moderately sensitive area is the largest area. Furthermore, the second largest region is made up of highly sensitive places. The highly susceptible area occupies the majority of the resulting vulnerable areas and is dispersed along the shoreline in the second scenario. The second-greatest area, however, is filled by moderately sensitive areas. According to the third scenario, which places the most emphasis on topography, there are four levels of susceptibility, ranging from low to very high. The sensitive zones of medium and high degree make up the majority of the city's vulnerable area.

According to the fourth scenario, which focuses on geology, the largest area in danger, which accounts for the majority of the land, is highly vulnerable. The fifth scenario, which places the most emphasis on metrological data, is very similar to the effect of topography, where the largest inferred areas of vulnerability are medium and high vulnerabilities. The last scenario, which focuses on shoreline, has the same effect as the topography and metrological data scenarios. However, as indicated in Table 4 and Fig. 6, the three scenarios differ in terms of vulnerability degrees and their locations.

## Discussion

According to Table 4, Scenario 2 has the highest risky areas compared to scenario 1, estimated at 1.12 km^2^. This scenario considers the actual weights of the indicators based on experts’ judgments. Because of the variable nature of indicators, it is impossible to balance the relative weights of all variables, and hence the conclusions derived from experts’ experiences are more accurate. By comparing the results obtained from the geology parameter scenario (Scenario 4) and the equal weights parameters scenario (Scenario 2), it came to a consensus on the locations of the region's high and highly susceptible spots.

Refereeing to the results of the four sub-model parameters priorities (i.e., Scenarios 3, 4, 5, and 6) the most critical parameter of climate change that causes highly vulnerable areas in the coastal zone is the region’s geology, which constitutes the largest risky area of 1.7 km^2^. As a result, when developing new urban communities in coastal cities, it is critical to first preserve the integrity of the region's geology while also considering other issues. This finding agrees with Culshaw and Price (2011), who claim that geology is the most essential component in urban development and regeneration. Geologic hazards are responsible for significant loss of life and property destruction. As a result, studying a region’s geology helps to manage geologically based hazards that affect people and institutions as they interact with their built and natural environments. This result is an answer to the research question about the degree of climate change indicators effects on vulnerable area formation, where each indicator has its effects according to its values and importance.

The second parameter affected by climate change is the topographical structure of the region, which constitutes the second-largest risky area at 0.81 km^2^. The results are normal outcomes since coastal areas with high slopes and low elevation are considered great danger areas. This result asserts that the steep slopes of coastal locations are regarded as one of the primary reasons that put these areas in great danger, such as storm surge flooding and inundation (McInnes et al., 2006; Torresan et al., 2008). Low elevation values are also associated with very risky locations that are regarded as most vulnerable to inundation and flooding due to their proximity to or below sea level. On the other hand, areas of high elevation have a strong potential to resist flooding caused by increasing sea levels and storm surges (Mani Murali et al., 2013).

The third one affected by climate change and its effect on the development of new communities is the metrological data parameter, which constitutes 0.7 km^2^ risky areas. This impact resulted from Al Alamein New City being an inactive city without significant human, industrial, or economic activity. As a result, it faces modest problems in temperature, sea level pressure, precipitation, dew point temperature, and wind during the simulation time. Furthermore, this weak significance is due to the fact that the smaller the scale, the more sea levels and vulnerability to coastal disasters is determined by variables other than climate (Bongarts Lebbe et al., 2021).

The degradation of the shoreline is the least important characteristic considered when building new urban communities since it covers the smallest risky area and is estimated to be 0.65 km^2^. Despite the critical relevance of the magnitude of the shoreline region's deterioration, this deterioration is the consequence of both the influence of metrological data on the region and the impact of human activities. If climate conditions are regulated while limiting the effect of human activities, shoreline deterioration will be reduced. The shoreline is considered one of the dynamic landforms that constitute the coastal environment, it changes due to accretion and erosion processes caused by sea level rise, wave action, and sediment transportation (Kaliraj et al., 2015). In addition, erosion and accretion are closely related to climate change, as are the nature of the geological formations on the coastline and the movement of waves (El-Said, 2020).

**Fig. 7:**
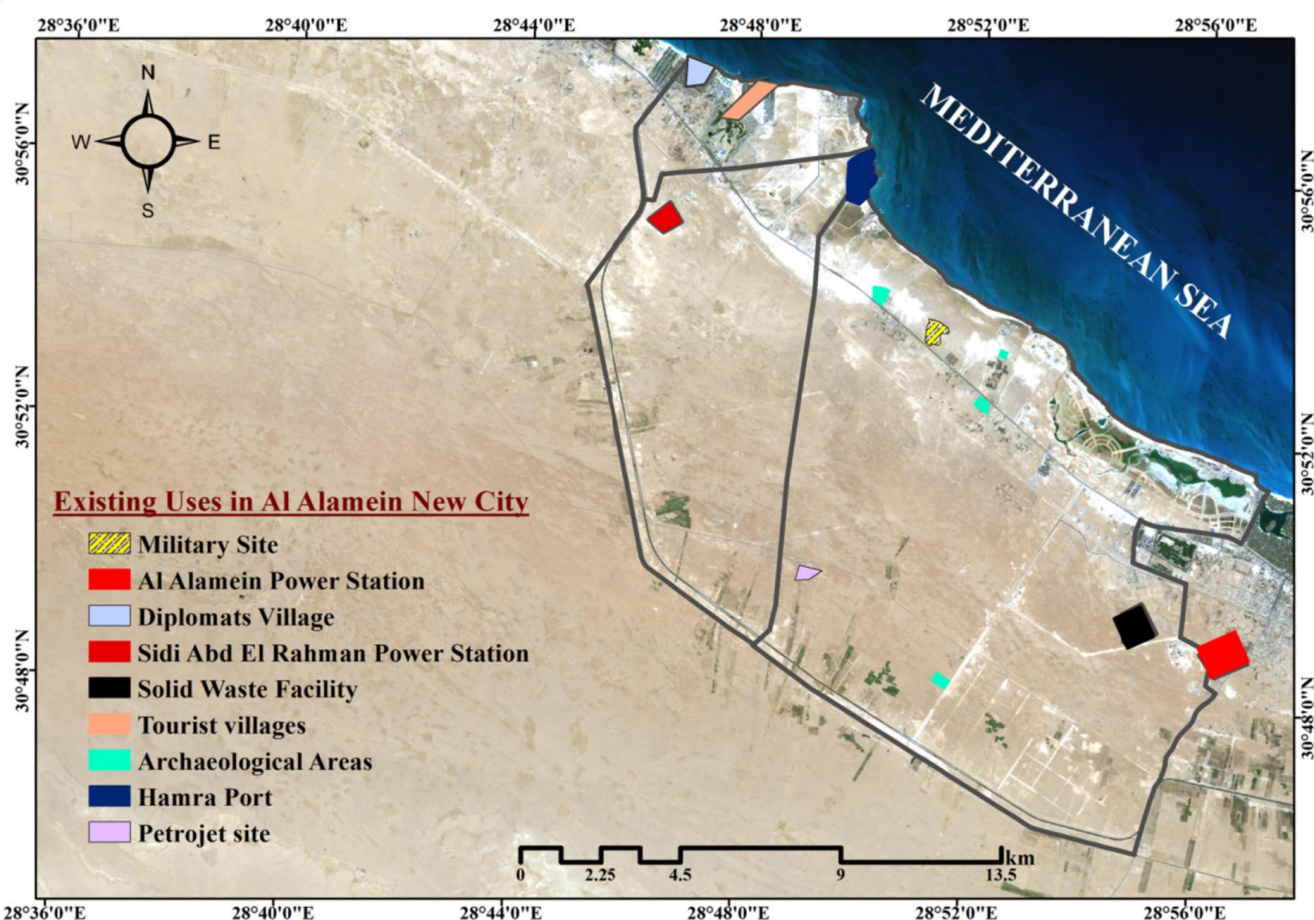
Existing uses within the boundaries of Al Alamein New City

**Fig. 8:**
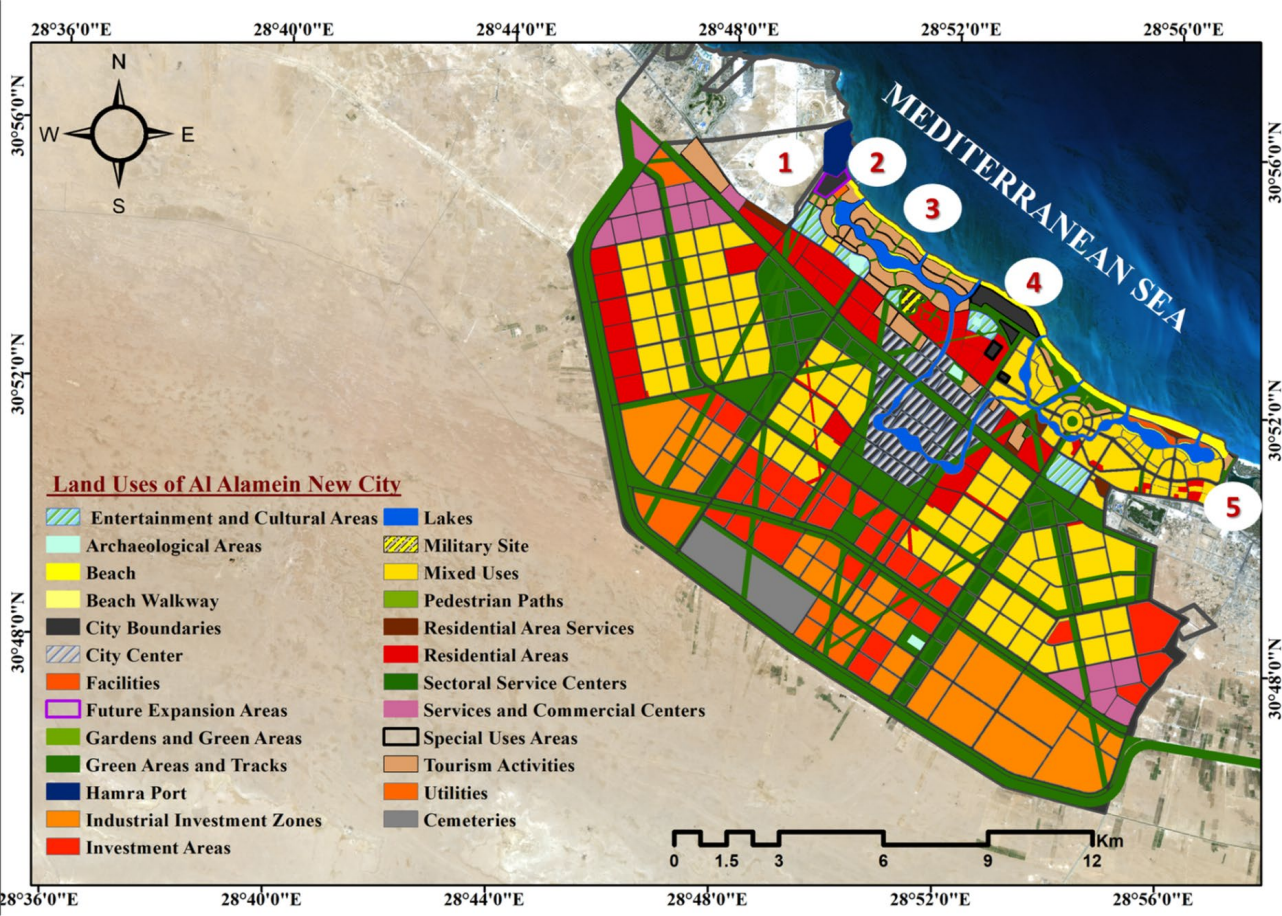
Vulnerable strategic locations in Al Alamein New City

According to its general strategic plan, Al Alamein New City was built to rejuvenate unused locations to attract investors, employees, and residents. tourist attractions, archaeological areas, industrial sites (Hamra port, Petrojet site, and solid waste facility), restricted sites, and utility stations are all existing uses inside the city boundaries, as shown in Fig. 7. However, the city’s general strategic plan is intended to include multiple sustainable uses of the land and existing uses to achieve the city’s strategic objectives, as shown in Fig. 8. By comparing the general strategic plan with the city’s coastal vulnerable locations and taking into consideration the prohibited activities in the coastal zone under Water Resources and Irrigation Law No. 147 of 2021, a buffer zone was created using a GIS environment at a distance of 200 m from the shoreline to the land to detect the uses located in the prohibited area as well as those located in highly vulnerable sites due to climatic changes. There are five strategic locations at risk: 1) Hamra port; 2) future expansion areas that may include a tourist and residential extension; 3) tourist activities in the North Coastal Road that include commercial services, conference centers, parks, museums, and sports activities to serve the tourist villages scattered along the coast to prolong the tourist season; 4) areas for special uses such as the establishment of a zone for technological industries in Al Alamein; and 5) tourism activities in East Marina Al Alamein as well. As a result of the hazards of climate change, the creation of these communities on the shoreline must be prohibited, necessitating an adequate adaptation plan to safeguard the city from numerous harmful consequences.

**Fig. 9:**
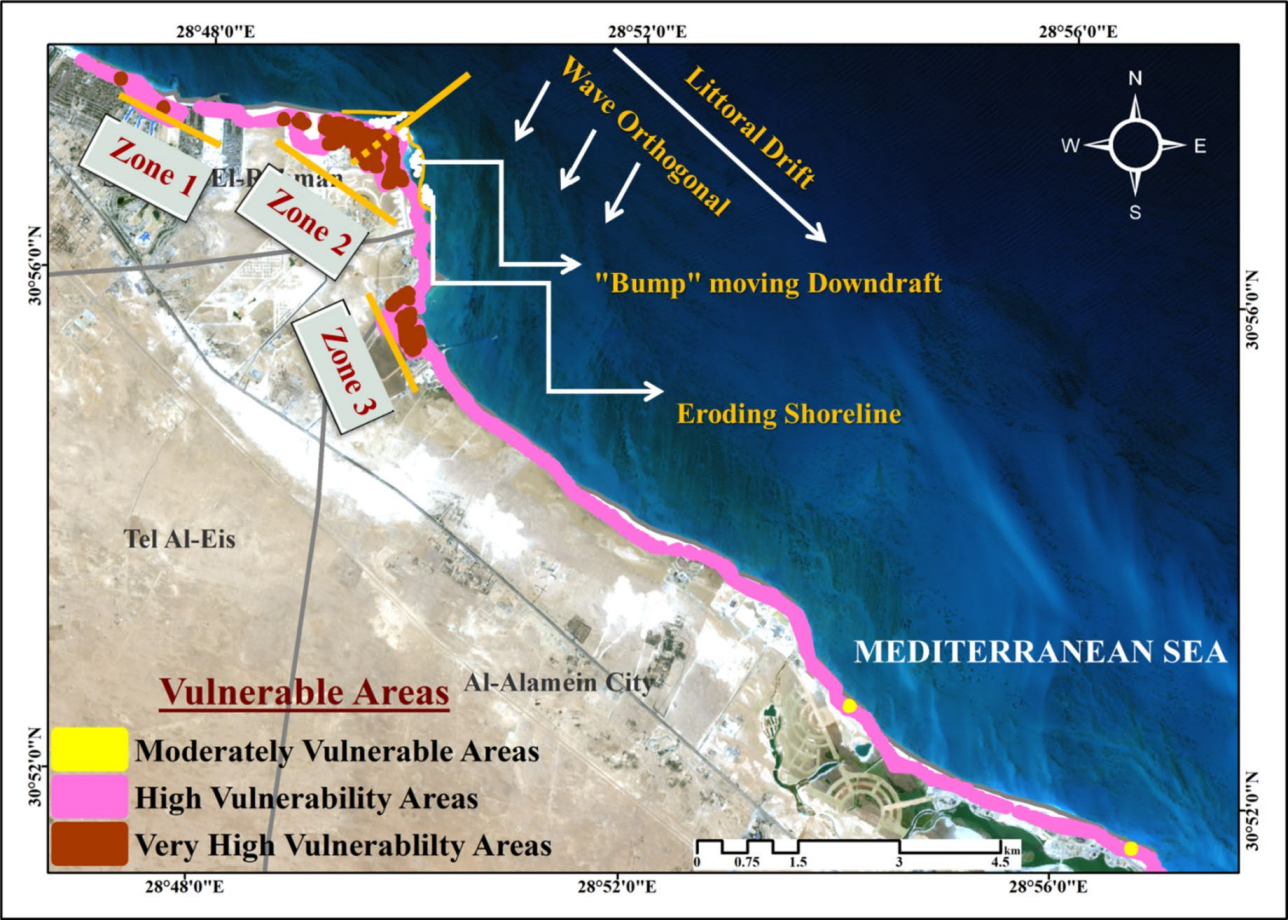
Hard protection effects on the shoreline of Al Alamein New City

According to research results, there is a need to include adaptation and mitigation measures in the planning of communities to safeguard the city against the destructive impacts of climate change and reduce future vulnerability along the shoreline. Figure 9 depicts the areas in Al Alamein new city, which are designated as zones 1, 2, and 3. These zones should have priority in protection because they contain important strategic locations, namely Hamra Mina and future expansion areas for tourist and residential communities. The breakwater hard protection system is the defensive device used to defend Al Alamein New City’s beaches. Because natural processes such as waves and artificial barriers around the shoreline interact complexly over time, relying on this system alone to safeguard the shoreline from climate change impacts, particularly SLR, has a negative long-term impact. The conclusion drawn from evaluating how these barriers affect the most vulnerable zones indicated earlier is that sand moves around the downdrift headland of breakwater barriers due to ongoing wave motion. As shown in Fig. 9, barriers that significantly reduce littoral drift, alongshore sediment movement and beach material erosion will also induce sediment retention behind the barriers. There will be more erosion and accretion regions along the shoreline due to the waves’ continued interactions with the barriers and the change in their intensity over time, which raises the danger of vulnerable areas (Barton & Brown, 2019). This is consistent with the results of El-Masry et al. (2022) study, which found that the existing adaptive measures in the El Hammam—EL Alamein area are insufficient to deal with future climate change, SLR, and other natural risks like storms and tsunamis. Therefore, this paper recommends developing a hybrid adaptation system that uses a combination of soft protection and hard infrastructure to provide effective and long-term adaptation to the effects of climate change in the study area. It has the ability to defend the shoreline against SLR, erosion, and other negative effects of climate change. In addition, it is more cost-effective in the long run than hard infrastructure alone (Bongarts Lebbe et al., 2021; Sutton-Grier et al., 2015). Choosing a combination of protection systems requires considering the balance of recreational access, a lifetime of systems, and budget constraints.

According to the advantages and disadvantages of the artificial and natural protection systems for the beaches listed in Appendices 1 and 2, a hybrid adaptation system can be recommended for the study area. The most viable options for protecting the Al Alamein New City's shoreline are using a combination of breakwaters as hard engineering protection and afforestation of coastal dunes as soft engineering protection. Breakwaters absorb wave energy during storm events, reducing the consequences of storm surge and coastal erosion. The chosen natural protection can overcome the erosion and accretion caused by these barriers. Coastal dunes have the potential to protect cities from storm surges and wave attacks. They offer a natural particulate barrier that erodes during storms, dissipating wave energy. Planted sand dunes help to improve the dune's capacity to resist erosion.

## Conclusions

Spatial vulnerability maps due to climate change impacts for Al Alamein New City have been generated according to the developed decision-making framework. The proposed framework overcomes the problem of recognizing the destructive consequences of climate change on coastal communities due to its ability to evaluate the studied parameters with their indicators in the formulation of risky vulnerable zones from a holistic perspective. The parameters include meteorological data, topographical structure, engineering geology, and shoreline. Vulnerable areas susceptible to these parameters are identified along the study coastline. These areas are located in three portions of the study area: Sidi Abd El-Rahman, Al Alamein City, and Tel Al-Eis. The risks involved range from modest to very high. Based on the relative relevance of each vulnerable parameter, the geology of the region is the most essential parameter influenced by climate change due to its propensity to form a highly sensitive area in the coastal zone. As a result, developing new urban settlements in coastal areas demands first conserving the integrity of the region's geology while also taking into account other challenges, such as coastal topography, meteorological data, and shoreline.

Although the proposed framework’s vulnerable areas range from low to very high, they must be carefully equipped to deal with future climatic changes and be suitable for the displacement of the neighboring region's citizens. To adapt to the long-term effects of climate change on coastal zones in a sustainable manner, a combination of soft and hard protective systems should be considered. The paper recommends a hybrid adaptation system for the examined area. It combines breakwaters as hard engineering protection and afforestation of coastal dunes as soft engineering protection to overcome current limitations of presently utilized protections such as downdraft erosion and interrupting natural processes.

## Figures and Tables

**Table 5:**
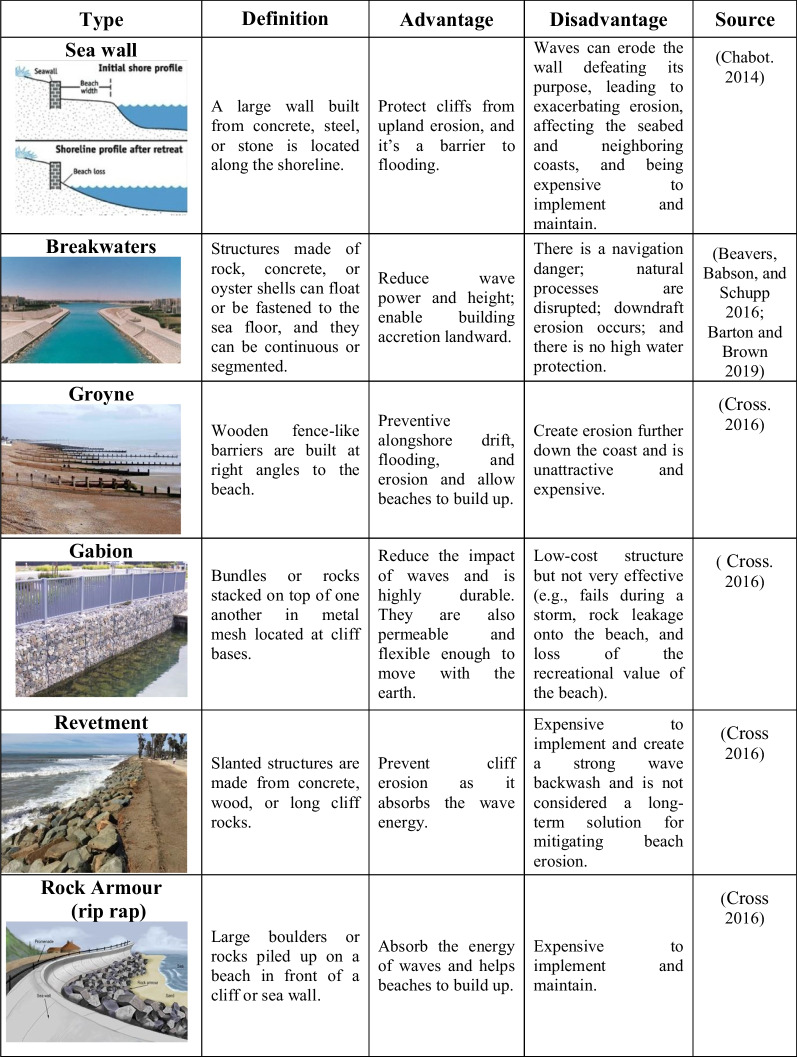
Hard engineering protection of coast

**Table 6:**
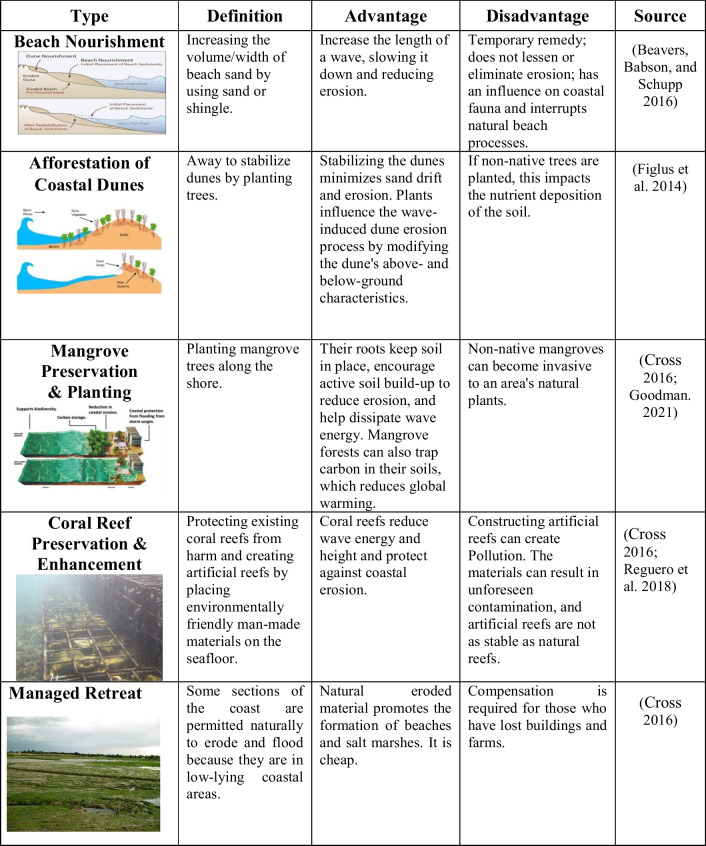
Soft engineering protection of coast

## Data Availability

Not applicable.

## Appendix 1

Table 5.

## Appendix 2

Table 6.

## References

[CR1] Allard, C. (2017). Regional economic outlook. Sub-Saharan Africa. Washington, D.C.: International Monetary Fund. Available at: https://books.google.com.eg/books?id=n18ZEAAAQBAJ&dq=Regional+economic+outlook.+Sub-Saharan+Africa+2017&lr=&source=gbs_navlinks_s

[CR2] Allipour Birgani R, Takian A, Djazayery A, Kianirad A, Pouraram H (2022). Climate Change and Food Security Prioritizing Indices: Applying Analytical Hierarchy Process (AHP) and Social Network Analysis (SNA). Sustainability.

[CR3] Arun PV (2013). A comparative analysis of different DEM interpolation methods. The Egyptian Journal of Remote Sensing and Space Science.

[CR4] Azab, S. (2022). Assessment of climate changes impact on coastal vulnerability for feasibility of developing new communities. Ph.D thesis, Faculty of Engineering, Cairo University. Available at: http://espsb.mans.edu.eg/eulc_v5/Libraries/Thesis/BrowseThesisPages.aspx?fn=PublicDrawThesis&BibID=12973592

[CR5] Bagheri M, Sulaiman WNA, Vaghefi N (2013). Application of geographic information system technique and analytical hierarchy process model for land-use suitability analysis on coastal area. Journal of Coastal Conservation.

[CR6] Bagheri M, Zaiton Ibrahim Z, Mansor S, Abd Manaf L, Akhir MF, Talaat WIAW, Beiranvand Pour A (2021). Application of Multi-Criteria Decision-Making Model and Expert Choice Software for Coastal City Vulnerability Evaluation. Urban Science.

[CR7] Barton, M., Brown, S. (2019). Shoreline Response to Littoral Drift Barriers. In: Finkl, C.W., Makowski, C. (eds) Encyclopedia of Coastal Science. Encyclopedia of Earth Sciences Series. Springer, Cham. 10.1007/978-3-319-93806-6_394

[CR8] Baučić, M., Ivić, M., Jovanović, N. & Bačić, S. (2019). Vulnerability analysis for the integrated coastal zone management plan of the City of Kaštela in Croatia. *The International Archives of the Photogrammetry, Remote Sensing and Spatial Information Sciences, 42*, pp. 59–63. Available at: https://isprs-archives.copernicus.org/articles/XLII-3-W8/59/2019/

[CR9] Beavers RL, Babson A, Schupp CA (2016). Coastal Adaptation Strategies Handbook: National Park Service Report 2016.

[CR10] Bongarts Lebbe, T., Rey-Valette, H., Chaumillon, É., Camus, G., Almar, R., Cazenave, A., Claudet, J., Rocle, N., Meur-Ferec, C., Viard, F. & Mercier, D. (2021). Designing coastal adaptation strategies to tackle sea level rise. *Frontiers in Marine Science, 8*, p. 1640. 10.3389/fmars.2021.740602

[CR11] Boulomytis, V. T. G., Zuffo, A. C. and Gireli, T. Z. (2015). Multi-criteria decision making for the assessment of coastal flood vulnerability. In World Environmental and Water Resources Congress 2015, (pp. 1248–1255). 10.1061/9780784479162.123

[CR12] Cabana, D., Rölfer, L., Evadzi, P. & Celliers, L., (2023). Enabling climate change adaptation in coastal systems: A systematic literature review. *Earth's Future, 11*(8), p.e2023EF003713. 10.1029/2023EF003713

[CR13] Chabot. W., 2014. 'Seawalls Kill Beach', Accessed May 25. https://coastalcare.org/2014/10/seawalls-kill-beaches-open-letters-by-warner-chabot-and-rob-young/.

[CR14] Chauhan, D., Thiyaharajan, M., Pandey, A., Singh, N., Singh, V., Sen, S. & Pandey, R. (2021). Climate change water vulnerability and adaptation mechanism in a Himalayan City, Nainital, India. *Environmental Science and Pollution Research*, pp. 1–18. 10.1007/s11356-021-15713-5 10.1007/s11356-021-15713-5 34331647

[CR15] Childs C (2004). Interpolating surfaces in ArcGIS spatial analyst. ArcUser, July-September.

[CR16] CoastAdapt. (2017). Overview of the impacts on our coast. Available from: https://coastadapt.com.au/overview-likely-climate-change-impacts-coast. Accessed 23 Mar 2021.

[CR17] Consultants A (2015). Al-Alamein New City (ANC), conceptual design.

[CR18] Cowen, D. J. (1990). GIS versus CAD versus DBMS: What are the differences? In Introductory readings in geographic information systems (1st ed., pp. 70–80). CRC Press. https://www.taylorfrancis.com/chapters/edit/10.1201/b12579-11/gis-versus-cad-versus-dbms-differences-david-cowen

[CR19] Cross. C. 2016. 'The difference between soft and hard engineering', Accessed May 25. https://www.theswimguide.org/2016/09/22/difference-soft-hard-engineering/.

[CR20] Culshaw MG, Price SJ (2011). The 2010 Hans Cloos lecture: The contribution of urban geology to the development, regeneration and conservation of cities. Bulletin of Engineering Geology and the Environment.

[CR21] El-Masry EA, El-Sayed MK, Awad MA, El-Sammak AA, Sabarouti MAE (2022). Vulnerability of tourism to climate change on the Mediterranean coastal area of El Hammam–EL Alamein. Egypt. Environment, Development and Sustainability.

[CR22] El-Said. M. 2020. Egypt to lose 1000 km of sandy coasts due to erosion. Retrieved December 25, 2021. Available at: https://uh.edu/~jbutler/physical/chapter7notes.html.

[CR23] El-Shahat S, El-Zafarany AM, El Seoud TA, Ghoniem SA (2021). Vulnerability assessment of African coasts to sea level rise using GIS and remote sensing. Environment, Development and Sustainability.

[CR24] Change, I.C . 2022. "Impacts, Adaptation, and Vulnerability." In.

[CR25] Figlus J, Sigren JM, Armitage AR, Tyler RC (2014). Erosion of vegetated coastal dunes. Coastal Engineering Proceedings.

[CR26] French PW (2004). The changing nature of, and approaches to, UK coastal management at the start of the twenty-first century. Geographical Journal.

[CR27] Gargiulo C, Battarra R, Tremiterra MR (2020). Coastal areas and climate change: A decision support tool for implementing adaptation measures. Land Use Policy.

[CR28] Goodman. E. 2021. 'Benefits of Mangroves - Flood Protection', Accessed May 25. https://www.theleafcharity.com/blog/benefits-of-mangroves-flood-protection.

[CR29] Haugen A, Bertolin C, Leijonhufvud G, Olstad T, Broström T (2018). A methodology for long-term monitoring of climate change impacts on historic buildings. Geosciences.

[CR30] Huang F, Liu D, Tan X, Wang J, Chen Y, He B (2011). Explorations of the implementation of a parallel IDW interpolation algorithm in a Linux cluster-based parallel GIS. Computers & Geosciences.

[CR31] Hutchinson, M. F. (1988). Calculation of hydrologically sound digital elevation models. In *Proceedings of the Third International Symposium on Spatial Data Handling, 133*, pp. 117–133. https://www.researchgate.net/publication/242529374_Calculation_of_Hydrologically_Sound_Digital_Elevation_Models

[CR32] IPCC. (1992). Global Climate Change and the rising challenge of the sea. Ministry of Transport, Public Works and Water Management, Directorate General Rijkswaterstaat, Tidal Waters Division.

[CR33] IPCC. (2001). Climate Change 2001: The Scientific Basis. Contribution of Working Group I to the Third Assessment Report of the Intergovernmental Panel on Climate Change. In J. T. Houghton, Y. Ding, D. J. Griggs, M. Noguer, P. J. van der Linden, X. Dai, K. Maskell, & C. A. Johnson (Eds.). Cambridge University Press, Cambridge, United Kingdom and New York, NY, USA, p. 881. https://www.ipcc.ch/report/ar3/wg1/

[CR34] IPCC. (2013). Climate Change 2013: The Physical Science Basis. Contribution of Working Group I to the Fifth Assessment Report of the Intergovernmental Panel on Climate Change. In T. F. Stocker, D. Qin, G. K. Plattner, M. Tignor, S. K. Allen, J. Boschung, A. Nauels, Y. Xia, V. Bex & P. M. Midgley (Eds.). Cambridge University Press, Cambridge, United Kingdom and New York, NY, USA, p. 1535. https://www.ipcc.ch/report/ar5/wg1/

[CR35] IPCC. (2014). Climate Change 2014: Synthesis Report. Contribution of Working Groups I, II and III to the Fifth Assessment Report of the Intergovernmental Panel on Climate Change. In Core Writing Team, R. K. Pachauri & L. A. Meyer (Eds.). IPCC, Geneva, Switzerland, p. 118. Available at: https://www.ipcc.ch/report/ar5/syr/

[CR36] Jiang H, Yu Y, Chen MM, Huang W (2021). The climate change vulnerability of China: Spatial evolution and driving factors. Environmental Science and Pollution Research.

[CR37] Kaliraj S, Chandrasekar N, Magesh NS (2015). Evaluation of coastal erosion and accretion processes along the southwest coast of Kanyakumari, Tamil Nadu using geospatial techniques. Arabian Journal of Geosciences.

[CR38] Kim Y, Chung ES (2013). Assessing climate change vulnerability with group multi-criteria decision making approaches. Climatic Change.

[CR39] Li, J., & Heap, A. D. (2008). A review of spatial interpolation methods for environmental scientists. Geoscience Australia, Record 23, p. 137. https://www.researchgate.net/profile/Jin-Li-74/publication/246546630_A_Review_of_Spatial_Interpolation_Methods_for_Environmental_Scientists/links/56f9ccb408ae95e8b6d40461/A-Review-of-Spatial-Interpolation-Methods-for-Environmental-Scientists.pdf

[CR40] Li, X., Rowley, R. J., Kostelnick, J. C. (2009). GIS analysis of global impacts from sea level rise. *Photogrammetric Engineering & Remote Sensing 75*, 807–818.

[CR41] Luo S, Cai F, Liu H, Lei G, Qi H, Su X (2015). Adaptive measures adopted for risk reduction of coastal erosion in the People's Republic of China. Ocean & Coastal Management.

[CR42] Maanan M, Maanan M, Rueff H, Adouk N, Zourarah B, Rhinane H (2018). Assess the human and environmental vulnerability for coastal hazard by using a multi-criteria decision analysis. Human and Ecological Risk Assessment: An International Journal.

[CR43] Mani Murali R, Ankita M, Amrita S, Vethamony P (2013). Coastal vulnerability assessment of Puducherry coast, India, using the analytical hierarchical process. Natural Hazards and Earth System Sciences.

[CR44] Marzouk M, Attia K, Azab S (2021). Assessment of coastal vulnerability to climate change impacts using GIS and remote sensing: A case study of Al-Alamein New City. Journal of Cleaner Production.

[CR45] McFadden L (2007). Governing coastal spaces: The case of disappearing science in integrated coastal zone management. Coastal Management.

[CR46] McInnes, R., Helen, F., & Jenny, J. (2006). Responding to the risks from Climate Change in coastal zones: A good practice guide. Isle of Wight Centre for the Coastal Environment. https://www.preventionweb.net/quick/38463

[CR47] NASA’s open data portal: Prediction Of Worldwide Energy Resources (POWER NASA). (2019). Available at: https://power.larc.nasa.gov/data-access-viewer/. Accessed 3 Jul 2019.

[CR48] National Authority for Remote Sensing & Space Sciences (NARSS). (2007). Updating Egypt's map of available building materials and existing and future industries Project, Egyptian General Petroleum Corporation, CONOCO-Coral, “The Geological Map of Egypt, Scale 1:500,000,” National Authority of Remote Sensing and Space Science, Egypt, Cairo, 1987.

[CR49] National Oceanic and Atmospheric Administration (NOAA). (2019). Climate Data online. National Climatic Data Center at National Center of Environmental Information. U.S. Department of Commerce. Available from: https://www.noaa.gov/. Accessed 20 Jul 2019.

[CR50] Nelson, S. A., 2018. Coastal Zones. Available at: https://www.tulane.edu/~sanelson/Natural_Disasters/coastalzones.htm.

[CR51] Palacios-Abrantes, J., Badhe, R., Bamford, A., Cheung, W.W., Foden, W., Frazão Santos, C., Grey, K.A., Kühn, N., Maciejewski, K., McGhie, H. and Midgley, G.F. (2022). Managing biodiversity in the Anthropocene: Discussing the Nature Futures Framework as a tool for adaptive decision-making for nature under climate change. *Sustainability Science*, pp. 1–17. 10.1007/s11625-022-01200-4

[CR52] Papari, G., Petkov, N. (2009). Reduced inverse distance weighting interpolation for painterly rendering. In: X. Jiang, & N. Petkov (Eds.), Computer Analysis of Images and Patterns. CAIP 2009. Lecture Notes in Computer Science, vol 5702. Springer, Berlin, Heidelberg. 10.1007/978-3-642-03767-2_62

[CR53] Rahman, B. (2018). Alarming environmental degradation: Preventive and mitigation measures. *Daily sun*. Available from: https://www.daily-sun.com/printversion/details/285434/Alarming-Environmental-Degradation:--Preventive-and-Mitigation-Measures. Accessed 13 Jan 2019.

[CR54] Rahmawan, G. A., Dhiauddin, R., Wisha, U. J., Gemilang, W. A., Syetiawan, A., Ambarwulan, W. & Rahadiati, A. (2022). Gis-Based assessment of coastal vulnerability in the Jatabek (Jakarta, Tangerang, Andbekasi) Region, Indonesia. *Geographia Technica, 17*(2). pp 84–96. 10.21163/GT_2022.172.08

[CR55] Rangel-Buitrago N, Neal WJ, Bonetti J, Anfuso G, de Jonge VN (2020). Vulnerability assessments as a tool for the coastal and marine hazards management: An overview. Ocean & Coastal Management.

[CR56] Reguero BG, Beck MW, Agostini VN, Kramer P, Hancock B (2018). Coral reefs for coastal protection: A new methodological approach and engineering case study in Grenada. Journal of Environmental Management.

[CR57] Roy P, Pal SC, Chakrabortty R, Chowdhuri I, Saha A, Shit M (2023). Effects of climate change and sea-level rise on coastal habitat: Vulnerability assessment, adaptation strategies and policy recommendations. Journal of Environmental Management.

[CR58] Saaty TL (1977). A scaling method for priorities in hierarchical structures. Journal of Mathematical Psychology.

[CR59] Saaty TL (1985). Decision making for leaders. IEEE Transactions on Systems, Man, and Cybernetics.

[CR60] Sahoo B, Bhaskaran PK (2018). Multi-hazard risk assessment of coastal vulnerability from tropical cyclones–A GIS based approach for the Odisha coast. Journal of Environmental Management.

[CR61] Sanuy M, Duo E, Jäger WS, Ciavola P, Jiménez JA (2018). Linking source with consequences of coastal storm impacts for climate change and risk reduction scenarios for Mediterranean sandy beaches. Natural Hazards and Earth System Sciences.

[CR62] Satta, A. (2014). An index-based method to assess vulnerabilities and risks of Mediterranean coastal zones to multiple hazards. Università Ca' Foscari Venezia. http://hdl.handle.net/10579/5594

[CR63] Sibson R (1981). A Brief Description of Nearest Neighbor Interpolation. Interpolating Multivariate Data. John Wiley & Sons, New York.

[CR64] Sutton-Grier AE, Wowk K, Bamford H (2015). Future of our coasts: The potential for natural and hybrid infrastructure to enhance the resilience of our coastal communities, economies and ecosystems. Environmental Science & Policy.

[CR65] Thirumurthy S, Jayanthi M, Samynathan M, Duraisamy M, Kabiraj S, Anbazhahan N (2022). Multi-criteria coastal environmental vulnerability assessment using analytic hierarchy process based uncertainty analysis integrated into GIS. Journal of Environmental Management.

[CR66] Torresan S, Critto A, Dalla Valle M, Harvey N, Marcomini A (2008). Assessing coastal vulnerability to climate change: Comparing segmentation at global and regional scales. Sustainability Science.

[CR67] VanZomeren, C., & Aeevedo-Mackey, D. (2019). A review of coastal vulnerability assessments: Definitions, components, and variables, environmental laboratory (U.S.). 10.21079/11681/33289

[CR68] Vieira LR, Vieira JG, Silva IMD, Barbieri E, Morgado F (2021). GIS models for vulnerability of coastal erosion assessment in a tropical protected area. ISPRS International Journal of Geo-Information.

[CR69] Wei P, Peng Y, Chen W (2021). Climate change vulnerability and key adaptation trajectory of the regional economic system. Discrete Dynamics in Nature and Society.

[CR70] Woodruff, S., Vitro, K. A., & BenDor, T. K. (2018). GIS and coastal vulnerability to Climate Change. *Comprehensive Geographic Information Systems* (pp. 236–257). Elsevier. 10.1016/b978-0-12-409548-9.09655-x

[CR71] World Meteorological Organization. (2017). WMO guidelines on the calculation of climate normals, WMO-No. 1203.

[CR72] Wunsch C, Stammer D (1997). Atmospheric loading and the oceanic “inverted barometer” effect. Reviews of Geophysics.

[CR73] Yannis G, Kopsacheili A, Dragomanovits A, Petraki V (2020). State-of-the-art review on multi-criteria decision-making in the transport sector. Journal of Traffic and Transportation Engineering (english Edition).

